# Leukemia cutis at onset of acute leukemia of ambiguous lineage

**DOI:** 10.1002/jha2.144

**Published:** 2020-11-30

**Authors:** Bernardo Lopez‐Andrade, Laura Lo Riso, Rafael Ramos, Vanesa Cunill, Julio F. Iglesias, Maria Antonia Duran Pastor

**Affiliations:** ^1^ Hematology department Hospital Universitario Son Espases Palma de Mallorca Spain; ^2^ Inmunology Department Hospital Universitario Son Espases Palma de Mallorca Spain; ^3^ Pathology Department Hospital Universitario Son Espases Palma de Mallorca Spain

A 50‐year‐old male consulted the emergency room due to the appearance of edema with a necrotic lesion on his face around the zygomatic area. He reported the lesion had appeared in the previous 3 weeks and had worsened despite topical antibiotic treatment. Several new brownish‐brown skin lesions, with a smooth surface, were also found on both lower extremities. They were not itchy or painful, and the patient referred to them as asymptomatic (Figure 1A and B). Pancytopenia in the blood test prompted a peripheral blood film, confirming the presence of 40% blast cells in the peripheral blood, with apparently two different populations: medium‐sized blasts, some with scant cytoplasm granulation suggestive of myeloblasts, and smaller blast cells with basophilic cytoplasm suggestive of lymphoblasts.

A skin biopsy of the lesions and a bone marrow aspirate (BMA) were performed. The skin biopsy/cultures excluded infection and confirmed skin and intravascular blast infiltration (Figure 1C and D). Expression of CD20, CD3, PAX5, MPO, CD30, Cd34, CD117, and TdT was negative, with a *Ki* 67 of 30% in the tissue sample.

BMA confirmed leukemic debut with 56% blasts (40% myeloid‐like morphology and 16% lymphoid‐like morphology, as seen in the peripheral smear (Figure 1E and F). Immunophenotype of the blasts reported CD45+ low intensity, CD34+, CD19 −/+ (20%), CD79a Cytoplasmic (20%), HLA‐DR+, CD117+, CD13+, CD33+, CD11b−, CD16−, CD14−, CD64−, CD10−, CD58−, CD66c−, MPO−, and cytoplasmic CD3 negative. A total of 45% of blasts (CD45+, CD34+) expressed B‐cell line (CD19+, CD79a+) and myeloid (CD13+ CD33+ CD117+) markers. MPO was negative (Figure 1G).

Karyotype 47,XY,+X was nonspecific and genes *NPM1*, *BCR‐ABL1*, and *KMT2A* were not mutated, while a mutation in *FLT3‐ITD* tandem duplication was confirmed with a high ratio.

Following the World Health Organization (WHO) 2017 criteria, taking into account the immunohistochemistry in the tissue sample and the bone marrow, the immunophenotype was classified as acute leukemia of ambiguous lineage, not otherwise specified (NOS), with leukemia cutis at presentation. There was some debate in the classification as if we followed the European group for immunological characterization of acute leukemias (EGIL) criteria; it would fit as a mixed phenotype acute leukemia (MPAL) except that MPO negativity makes it difficult to classify as MPAL, and the WHO criteria are more widely accepted.

The patient started induction therapy, with disappearance of the skin lesiyons.

Leukemia cutis, or skin infiltration by leukemic cells, is an uncommon phenomenon that may precede the development of systemic leukemia, while acute leukemia of ambiguous lineage NOS is rare and its incidence is unknown. Both represent a diagnostic challenge with a poor overall prognosis.

**FIGURE 1:**
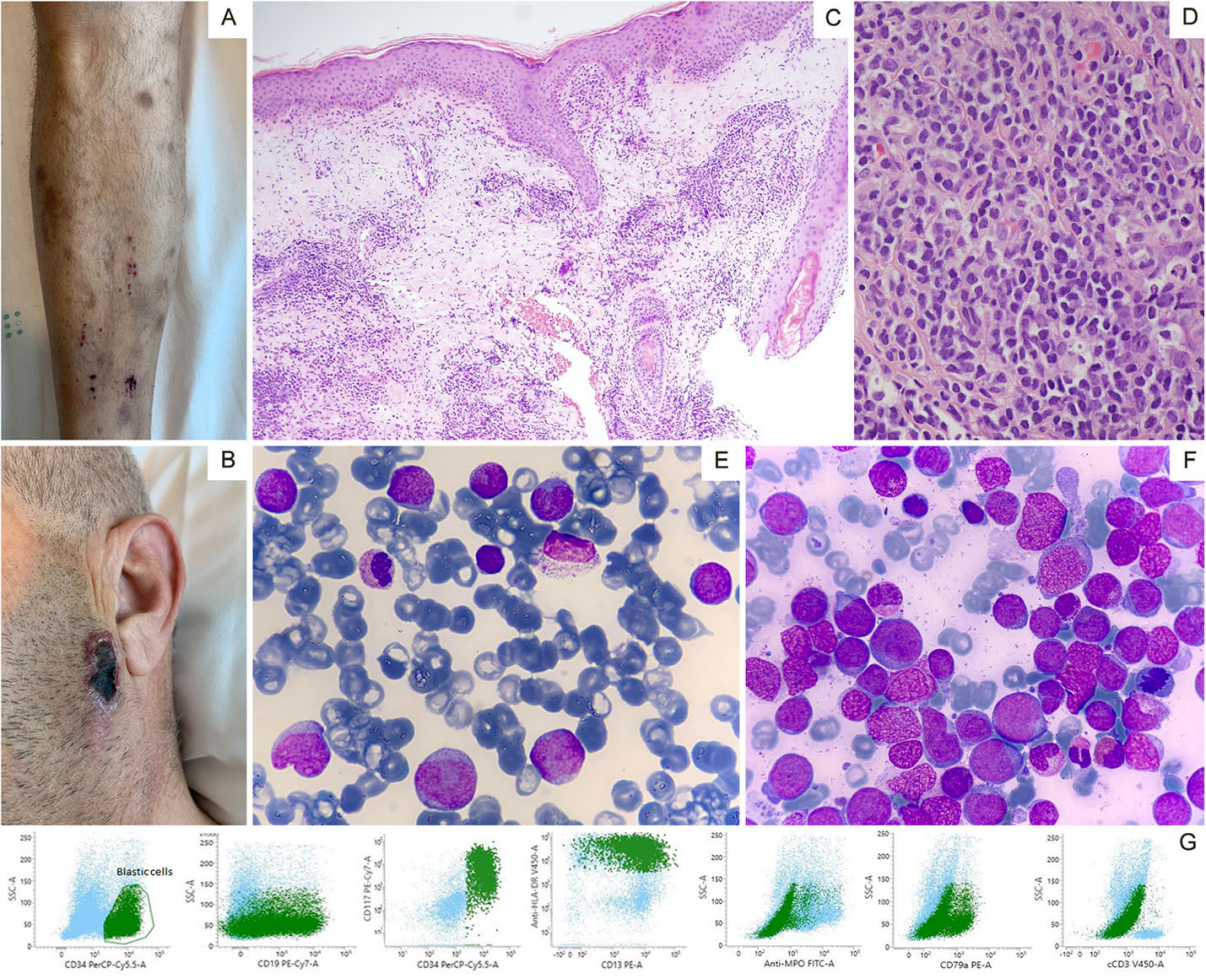
1A: Brown Skin lesions in the leg. 1B: Zygomatic area necrotic lesion. 1C: Subcutaneous infiltration by leukemic cells (Grunwald Giemsa 10x) 1D: Blast infiltration of subcutaneous tissue and vascular capillary. 1E: Peripheral blood film, Grunwald Giemsa 50x oil, different sized blasts. Small‐sized with blue cytoplasm and medium‐sized blasts. 1F: Bone marrow aspirate, Grunwald Giemsa 100x oil. Blast infiltration by different‐sized blast population. 1G: Bone marrow aspirate Immunophenotype.

## CONFLICT OF INTEREST

All the authors have no conflict of interest.

## PATIENT CONSENT STATEMENT

Written patient consent was obtained from the patient.

## Data Availability

The data reported in this study are available on request from the corresponding author. The data are not publicly available due to privacy or ethical restrictions.

